# Lignans, Amides, and Saponins from *Haplophyllum tuberculatum* and Their Antiprotozoal Activity

**DOI:** 10.3390/molecules25122825

**Published:** 2020-06-19

**Authors:** Abdelhalim Babiker Mahmoud, Ombeline Danton, Marcel Kaiser, Sohee Han, Aitor Moreno, Shereen Abd Algaffar, Sami Khalid, Won Keun Oh, Matthias Hamburger, Pascal Mäser

**Affiliations:** 1 Swiss Tropical and Public Health Institute, 4002 Basel, Switzerland; marcel.kaiser@swisstph.ch; 2 Faculty of Science, University of Basel, 4001 Basel, Switzerland; ombeline.danton@unibas.ch (O.D.); matthias.hamburger@unibas.ch (M.H.); 3 Faculty of Pharmacy, University of Khartoum, 11111 Khartoum, Sudan; khalidseek@hotmail.com; 4 Korea Bioactive Natural Material Bank, College of Pharmacy, Seoul National University, Seoul 08826, Korea; sohee.hn@snu.ac.kr (S.H.); wkoh1@snu.ac.kr (W.K.O.); 5 Bruker BioSpin, 8117 Fällanden, Switzerland; Aitor.Moreno@bruker.com; 6 Faculty of Pharmacy, University of Science and Technology, 14411 Omdurman, Sudan; phd_sh086@hotmail.com

**Keywords:** HPLC-Activity profiling, *Leishmania*, *Plasmodium*, *Trypanosoma*, *Haplophyllum tuberculatum*, lignans, cinnamoylphenethyl amides, steroid saponins

## Abstract

A screening of Sudanese medicinal plants for antiprotozoal activities revealed that the chloroform and water fractions of the ethanolic root extract of *Haplophyllum tuberculatum* exhibited appreciable bioactivity against *Leishmania donovani*. The antileishmanial activity was tracked by HPLC-based activity profiling, and eight compounds were isolated from the chloroform fraction. These included lignans tetrahydrofuroguaiacin B (**1**), nectandrin B (**2**), furoguaiaoxidin (**7**), and 3,3′-dimethoxy-4,4′-dihydroxylignan-9-ol (**10**), and four cinnamoylphenethyl amides, namely dihydro-feruloyltyramine (**5**), (*E*)-*N*-feruloyltyramine (**6**), *N*,*N*′-diferuloylputrescine (**8**), and 7′-ethoxy-feruloyltyramine (**9**). The water fraction yielded steroid saponins **11**–**13**. Compounds **1**, **2**, and **5**–**13** are reported for the first time from *Haplophyllum* species and the family Rutaceae. The antiprotozoal activity of the compounds plus two stereoisomeric tetrahydrofuran lignans—fragransin B_2_ (**3**) and fragransin B_1_ (**4**)—was determined against *Leishmania donovani* amastigotes, *Plasmodium falciparum,* and *Trypanosoma brucei rhodesiense* bloodstream forms, along with their cytotoxicity to rat myoblast L6 cells. Nectandrin B (**2**) exhibited the highest activity against *L. donovani* (IC_50_ 4.5 µM) and the highest selectivity index (25.5).

## 1. Introduction

Neglected tropical diseases (NTDs) are a group of infectious diseases that are prevalent in tropical and sub-tropical developing countries. NTDs are strongly associated with poverty, and of high socio-economic impact. NTDs account for 48 million disability-adjusted life years (DALYs) and 152,000 deaths per year [1,2]. The NTD leishmaniasis, caused by *Leishmania* spp., imposes a global burden of 3.3 million DALYS and 51,600 annual deaths [1,2]. There is no vaccine, and current drugs are problematic given their serious adverse effects and the emergence of drug-resistant parasites [3]. There is one efficient and safe drug, AmBisome, a liposomal formulation of amphotericin B [4]. However, the high price of the drug and the need of an uninterrupted cold chain for delivery severely limits its use. In Eastern Africa, a high-burden region of visceral leishmaniasis [5], sodium stibogluconate is still the mainstay of leishmaniasis chemotherapy [3]. This pentavalent antimonial can cause hepatotoxicity and cardiotoxicity [6]. Thus, there is an urgent need for the development of new, efficacious, safe, and cost-effective drugs for the treatment of leishmaniasis and other diseases caused by kinetoplastid parasites [7,8].

A library of Sudanese medicinal plants traditionally used as anti-infectives was screened for antiprotozoal activity against *Leishmania donovani, Trypanosoma brucei rhodesiense*, *Trypanosoma cruzi*, and *Plasmodium falciparum.* One of the most promising hits was *Haplophyllum tuberculatum* (Forssk.) A. Juss. (Rutaceae). Chloroform and aqueous fractions obtained by partitioning of an ethanolic extract from roots of the plant were active against *L. donovani* and *P. falciparum* (˃85% growth inhibition at 10 µg/mL) [9].

*Haplophyllum tuberculatum* is a perennial herb distributed throughout North Africa and the Middle East. In Sudan, the plant is locally known as *Haza,* and the aerial parts have been used traditionally to treat malaria, asthma, kidney diseases, gynecological and bowel disorders [10,11]. Anti-inflammatory, antioxidant, antibacterial, and antifungal activities have been reported [10,11,12], and alkaloids, flavonoids, coumarins and lignans have been identified [13,14,15]. The methanolic extract of the aerial part and roots of *H. tuberculatum* possessed activity against *P. falciparum* [16], and Justicidin A was found active [17]. The essential oil from the leaves had antileishmanial activity [18]. Justicidin B, a lignan isolated from the leaves, showed trypanocidal activity [19].

The aim of this project was a systematic evaluation of the antiparasitic activities of *H. tuberculatum* and the identification of the bioactive compounds by means of HPLC-based activity profiling. 

## 2. Results and Discussion

### 2.1. Extraction and HPLC-based Activity Profiling

**Figure 1:**
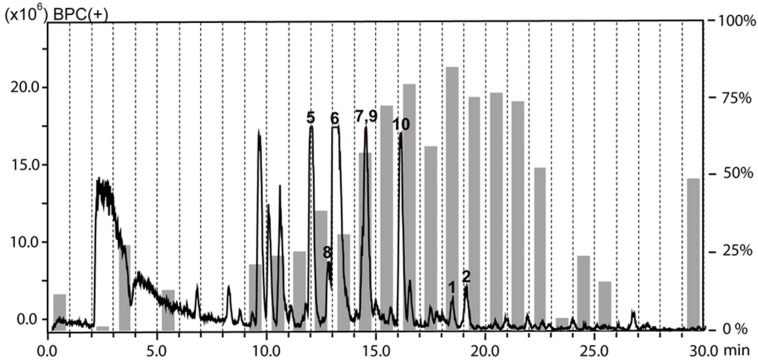
HPLC-based activity profiling of the chloroform fraction against axenic amastigotes of *L. donovani.* The ESIMS (base peak chromatogram in positive ion mode) from a separation of 300 μg of fraction is shown. For each microfraction the activity is expressed as percent growth inhibition in comparison to untreated cultures (grey bars). Bold numbers in the chromatogram refer to compounds **1**, **2**, and **5**–**10**.

**Figure 2:**
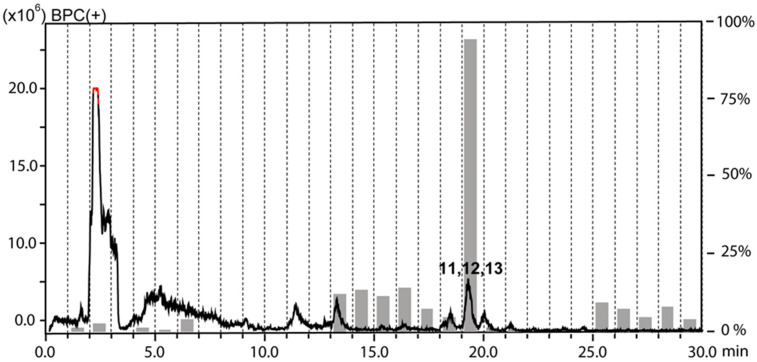
HPLC-based activity profiling of the water fraction against axenic amastigotes of *L. donovani*. The ESIMS (base peak chromatogram in positive ion mode) from a separation of 300 μg of fraction is shown. For each microfraction the activity is expressed as percent growth inhibition in comparison to untreated cultures (grey bars). Bold numbers in the chromatogram refer to compounds **11**–**13**.

The chloroform and the water fractions from roots of *H. tuberculatum* had been previously found to be active against *L. donovani* when tested at 10 µg/mL [9]. The antileishmanial activity was tracked by HPLC-based activity profiling, a procedure combining time-based microfractionation with bioactivity testing [20]. One-minute microfractions were collected and tested for growth inhibition of *L. donovani* axenic amastigotes. The HPLC-ESIMS traces (base peak chromatograms in positive ion mode) overlaid with the antileishmanial activity of microfractions are shown for the chloroform (Figure 1) and the water fraction (Figure 2). Pronounced antileishmanial activity (˃50% growth inhibition) was found in the chloroform fraction in the time window of 16–21 min, and moderate activity (30–40% growth inhibition) in the window of 12–15 min. For the water fraction, the antileishmanial activity was confined to a narrow time window between 19–20 min.

### 2.2. Compound Isolation and Structure Elucidation

**Figure 3:**
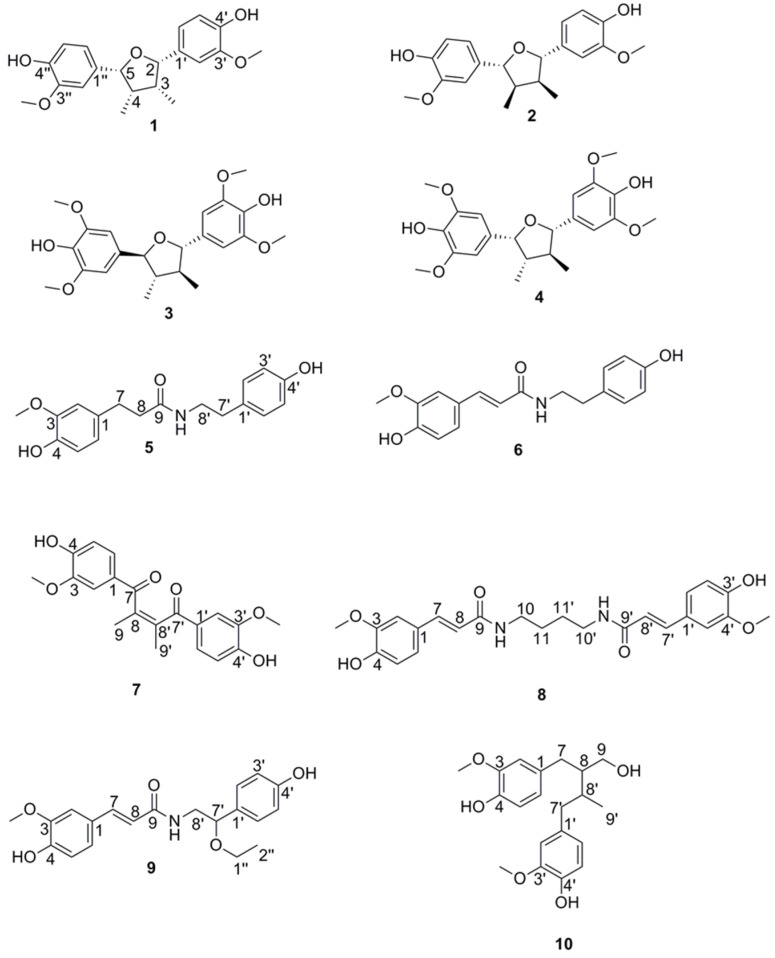
Chemical Structures of tetrahydrofuroguaiacin B (**1**), nectandrin B (**2**), fragransin B_2_ (**3**), fragransin B_1_ (**4**), dihydro-feruloyltyramine (**5**), (*E*)-*N*-feruloyltyramine (**6**), furoguaiaoxidin (**7**), *N*,*N*′-diferuloylputrescine (**8**), 7′-ethoxy-feruloyltyramine (**9**), and 3,3′-dimethoxy-4,4′-dihydroxylignan-9-ol (**10**).

Preparative separation by MPLC of the chloroform fraction on a C_18_ cartridge yielded 13 subfractions (A-M). HPLC-PDA-MS analysis showed that subfractions B and C contained peaks from the active time window. Further purification by semipreparative RP-HPLC afforded compounds **1** and **2** from subfraction B. Based on NMR data (Table S1, Supporting Information), the two compounds were identified as the known lignans tetrahydrofuroguaiacin B (**1**) and nectandrin B (**2**) (Figure 3) [21,22,23]. Semipreparative RP-HPLC of subfraction C afforded compounds **5**–**10.** By means of 1D and 2D NMR data (Tables S2 and S3, Supporting Information), these were identified as four cinnamoylphenethyl amides, namely dihydro-feruloyltyramine (**5**) [24], (*E*)-*N*-feruloyltyramine (**6**) [25], *N*,*N*′-diferuloylputrescine (**8**) [26], and 7′-ethoxy-feruloyltyramine (**9**) [27], and two lignans, furoguaiaoxidin (**7**) [23] and 3,3′-dimethoxy-4,4′-dihydroxylignan-9-ol (**10**) [28] (Figure 3). Compounds **9** and **10** showed optical rotation close to zero, and no cotton effects in the ECD spectra (Figures S1 and S2, Supporting Information). From a comparison with published data for the two compounds, we conclude that they mostly likely were racemic mixtures. 

Preparative RP chromatography by MPLC of the water fraction on a C_18_ cartridge yielded 15 subfractions (A-O), whereby subfractions J and K contained the peaks from the active time window of the activity profile (Figure 2). Semipreparative HPLC on a Hilic column afforded inseparable mixtures of **11** and **12** from subfraction J, and **11** and **13** from subfraction K. The HRESIMS spectrum of **11** exhibited a sodium adduct ion (*m/z* 1039.5083 [M + Na]^+^, calcd for C_50_H_80_O_21_Na^+^ 1039.5085) indicative of a molecular formula of C_50_H_80_O_21_. The NMR data (Table S4, Supporting Information) pointed to a steroidal saponin consisting of aglycon and four sugars. A comparison of ^13^C chemical shifts with literature data identified the aglycon as yamogenin [29]. This was corroborated by a detailed analysis of the 2D NMR spectra, in particular by cross peaks in the ROESY spectrum between H-1β (δ_H_ 1.78), H_3_-19 (δ_H_ 0.94), H-8 (δ_H_ 1.54), H_3_-18 (δ_H_ 0.72), and H-20 (δ_H_ 1.75), between H-1α (δ_H_ 0.96) and H-3 (δ_H_ 3.46), and between H_3_-21 (δ_H_ 0.92), H-17 (δ_H_ 1.65), H-16 (δ_H_ 4.27) and H-14 (δ_H_ 1.07). After hydrolysis and derivatization the sugars were identified as ᴅ -glucose, ʟ -rhamnose, and ᴅ -xylose [30]. The interglycosidic linkages and the attachment position of the sugar chain at the aglycon were established by HMBC correlations (Figures S3 and S4, Supporting Information) from δ_H_ 4.43 (1H, d, *J* = 7.5 Hz, H-1-Glc1) to δc 76.9 (C-3), from δ_H_ 5.04 (1H, br s, H-1-Rha1) to δc 76.4 (C-2-Glc1), from δ_H_ 4.34 (1H, d, *J* = 7.5 Hz, H-1-Glc2) to δc 81.1 (C-4-Glc1), and from δ_H_ 4.38 (1H, d, *J* = 7.1 Hz, H-1-Xyl) to δc 86.4 (C-3-Glc2). saponin **11** was identified as (*3*S,*20*S,*22R*,*25*S)-spirost-5-en-3-yl-(β-ᴅ-xylopyranosyl-(1→3)-β-d-glucopyranosyl-(1→4) [α-l-rhamnopyranosyl-(1→2)]-β-d-glucopyranoside [31].

**Figure 4:**
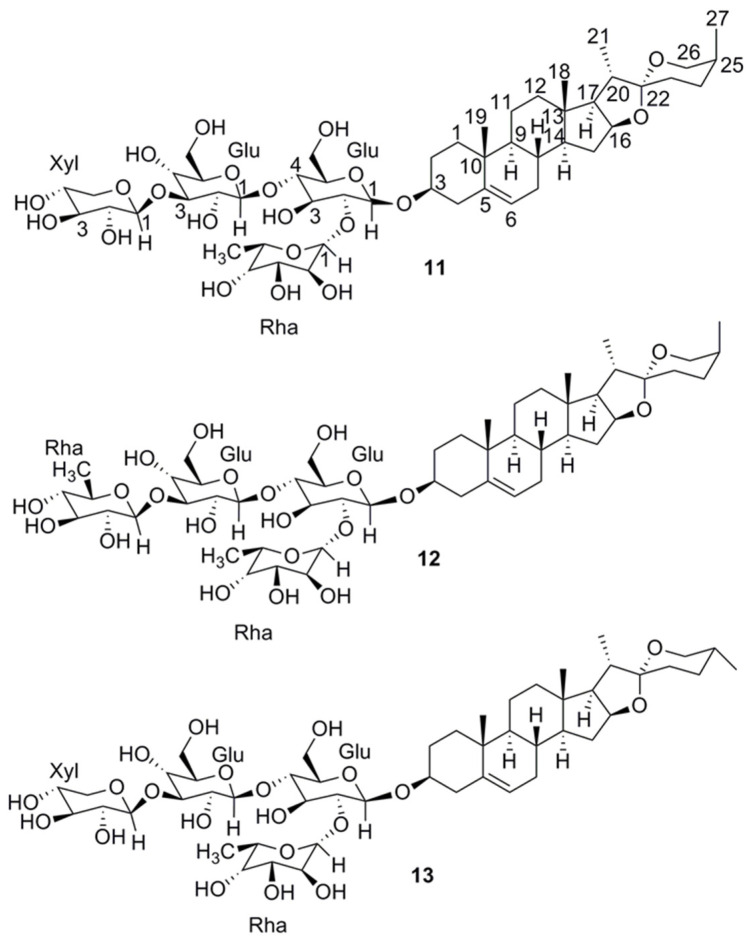
Chemical structures of (*3*S,*20*S,*22R*,*25*S)-spirost-5-en-3-yl-(β-d-xylopyranosyl-(1→3)-β-d-glucopyranosyl-(1→4) [α-l-rhamnopyranosyl-(1→2)]-β-d-glucopyranoside (**11**), (*3*S,*20*S,*22R*,*25*S)- spirost-5-en-3-yl-(β-d-rhamnopyranosyl-(1→3)-β-d-glucopyranosyl-(1→4)[α-l-rhamnopyranosyl-(1→2)]-β-d-glucopyranoside (**12**), and (*3*S,*20*S,*22*R,*25*R)-spirost-5-en-3-yl-(β-d-xylopyranosyl-(1→3)-β-d-glucopyranosyl-(1→4)[α-l-rhamnopyranosyl-(1→2)]-β-d-glucopyranoside (**13**).

Compound **12** had a molecular formula of C_51_H_82_O_21_ (HRESIMS data *m/z* 1053.5233 [M + Na]^+^, calcd for C_51_H_82_O_21_Na^+^ 1053.5241). As for compound **11**, NMR data (Table S4, Supporting Information) indicated yamogenin bearing four sugars. The only difference was in the presence of a rhamnopyranose instead of a xylopyranose (Figures S3 and S4, Supporting Information). Thus, saponin **12** was identified as (*3*S,*20*S,*22R*,*25*S)- spirost-5-en-3-yl-(β-d-rhamnopyranosyl-(1→3)-β-d-glucopyranosyl-(1→4)[α-l-rhamnopyranosyl-(1→2)]-β-d-glucopyranoside [32]. Compound **13** had a molecular formula of C_50_H_80_O_21_ (*m/z* 1039.5072 [M + Na]^+^, calcd for C_50_H_80_O_21_Na^+^ 1039.5085). Based on the NMR data and especially the carbon chemical shifts (Table S4, Supporting Information), the aglycone was identified as diosgenin [29]. The sugar moiety was identical to that in saponin **11** (Figures S5 and S6, Supporting Information). Compound **13** was therefore identified as (*3*S,*20*S,*22*R,*25*R)-spirost-5-en-3-yl-(β-d-xylopyranosyl-(1→3)-β-d-glucopyranosyl-(1→4)[α-l-rhamnopyranosyl-(1→2)]-β-d-glucopyranoside [31] (Figure 4). It is interesting to note that **11** and **13** are diastereoisomers with the C-25 methyl group in axial or equatorial orientation, respectively.

### 2.3. Comparison to Previously Reported Compounds

The two tetrahydrofuran-type lignans **1** and **2** were previously reported from different Myristicaceae, Elaeagnaceae, Poaceae, and Piperaceae species [22,33].

Arylnaphthalen-type lignans had been identified in aerial part and roots of *H. tuberculatum* [34,35], but tetrahydrofuran-type lignans are reported here for the first time from *Haplophyllum* species. 

Cinnamoylphenethyl amides such as **5**, **6**, **8** and **9** have been reported from over 30 families of flowering plants [36]. Amides **5** and **6** have been previously reported from different species of Annonaceae [24,37,38] and Lauraceae [39,40]. Compound **6** was also found in Papaveraceae [41], Cannabaceae [42], Solanaceae [25,43], and Portulacaceae [44,45], while **9** was identified in Portulacaceae [44,45] and Cactaceae [27]. Amide **7** has been previously reported from *Guaiacum officinale* [23], while compound **8** has been found in *Tribulus terrestris* [46], *Peltophorum pterocarpum* [47], and *Zea mays* [48]. Amide **10** has been previously identified in *Schisandra bicolor* var. *tuberculata* [28].

Saponins **11**–**13** have been previously isolated, together with a series of similar compounds, as molluscicidal saponins from the Sudanese medicinal plant *Balanites aegyptiaca* (Zygophyllaceae) [31,32,49]. It can be assumed that saponins **11**–**13** contributed to the molluscicidal activity that has been described for *H. tuberculatum* [50]. 

To the best of our knowledge, compounds **1**, **2**, and **5**–**13** have been isolated here for the first time not only from *Haplophyllum* species but also from plants of the Rutaceae family.

### 2.4. Biological Testing

Compounds **1**–**10** were tested for their in vitro activities against the following protozoan parasites: *Leishmania donovani* (MHOM/ET/67/L82) axenic amastigotes, *Plasmodium falciparum* (NF54) proliferative erythrocytic stages, and *Trypanosoma brucei rhodesiense* (STIB 900) bloodstream forms (Table 1). In parallel, cytotoxicity of these compounds in rat skeletal myoblasts (L-6 cells) was determined in order to obtain an initial assessment of their selectivity. The results are reported in Table 1. Subfractions **J** and **K** from the aqueous fraction containing steroidal saponins **11** and **12** in **J**, and **11** and **13** in **K**, exhibited IC_50_ ˂ 8 µg/mL across all parasites and a selectivity index ˂2, indicative of general cytotoxicity.

#### 2.4.1. Activity against Leishmania Donovani Axenic Amastigotes

Nectandrin B (**2**) was the most active (IC_50_ of 5 µM) and the most selective (SI 26) of all compounds tested. This finding contrasts with a previous report on nectandrin B being inactive against *L. donovani* [51]. The discrepancy may come from the fact that, in the aforementioned study, nectandrin B was tested against the promastigote form of the parasite (i.e., the proliferative form in the gut of the sandfly vector) while we tested against the amastigote form (i.e., the proliferative form in the mammalian host). The antileishmanial activity cannot be ascribed to any particular mode of action at this point. In general, some tetrahydrofuran lignans have been reported to inhibit trypanothione reductase [52], an enzyme of a thiol-redox system that is unique to trypanosomatid protozoa and essential for their survival [53,54]. Compared to **2**, tetrahydrofuroguaiacin B (**1**) was less active and more toxic. The two compounds only differ in their stereochemistry at the tetrahydrofuran ring, which therefore appears to play a crucial role in the antileishmanial activity of such lignans. To better understand the role of the stereochemistry at the central ring and the contribution of substituents at the aromatic rings, structurally related lignans **3** and **4** that had been previously reported from *Myristica fragrans* Houtt. [21,22] were also included in the testing. 

However, both compounds were significantly less active than nectrandrin B (**2**). Unfortunately, the activity of nectandrin B (**2**) against *L. donovani* was lost in the intracellular amastigote assay tested up to a concentration of 30 μM.

#### 2.4.2. Activity against Plasmodium Falciparum

Lignans **2** and **10** were the most active and least toxic compounds (IC_50_ 9–9.5 µM, SI 12–14). Compound **10** was selectively active against *P. falciparum*. Compared to **2**, fragransin B_1_ (**4**) (IC_50_ 17 µM, SI 8), was less active against *P. falciparum*, but was slightly more active and less cytotoxic than its stereoisomer fragransin B_2_ (**3**). Amides **8** (IC_50_ 30.6 µM, SI 4.8), and **9** (IC_50_ 30.4 µM, SI 6.8) showed comparable activity, while the congener **5** was the least active among all tested compounds.

A wide spectrum of biological activities of cinnamoylphenethyl amides has been described, including antiproliferative [55], antibacterial [56], antifungal [57] and antioxidant activities [58]. To the best of our knowledge, this is the first report of antileishmanial and antiplasmodial activities of such compounds.

#### 2.4.3. Activity against Trypanosoma Brucei Rhodesiense

Of all compounds tested, diferuloylputrescine (**8**) exhibited the highest activity against *T. b. rhodesiense* and the highest selectivity (IC_50_ 19 µM, SI 8). The lignan **10** showed a lower activity and selectivity index (IC_50_ 28 µM and SI 5), while **5** and **6** were the least active among all tested compounds. None of the tetrahydrofuran lignans **1**–**4** exhibited significant activity against *T. b. rhodesiense.*

## 3. Materials and Methods

### 3.1. General Experimental Procedures

HPLC grade solvents from Sigma-Aldrich (St. Louis, MO, USA), and Macron Fine Chemicals (Avantor Performance Materials, Phillipsburg, NJ, USA), and ultrapure water from a Milli-Q water purification system (Merck Millipore, Darmstadt, Germany) were used for HPLC separations. For extraction and preparative separation, technical grade solvents (Scharlab S. L., Barcelona, Spain) were used after distillation. Silica gel 60 F_254_ coated aluminum TLC plates were obtained from Merck (Darmstadt, Germany).

Optical rotation was measured in methanol on a JASCO P-2000 digital polarimeter (Tokyo, Japan) equipped with a sodium lamp (589 nm) and a temperature-controlled microcell (10 cm). UV and ECD spectra were recorded in methanol on a Chirascan CD spectrometer (Applied Photophysics, Leatherhead, UK) using 110 QS 1 mm path precision cells (Hellma Analytics, Müllheim, Germany). NMR spectra of compounds **1**, **2**, and **5**–**10** were recorded on a Bruker AVANCE III 500 MHz spectrometer (Billerica, CA, USA) operating at 500.13 MHz for ^1^H and 125.77 MHz for ^13^C. ^1^H, COSY, HSQC, HMBC, and NOESY spectra were measured at 23 °C in a 1-mm TXI probe with a z-gradient. The sample volume was 10 µL. NMR spectra of **11**–**13** were recorded on a Bruker AVANCE NEO 600 MHz spectrometer operating at 600.18 MHz for ^1^H and 150.92 MHz for ^13^C with an inverse 1.7 mm TCI micro-cryoprobe (30 µL sample volume) at 23 °C. This cryogenically cooled probe delivered a 4-fold gain of mass sensitivity over the 1 mm TXI room-temperature probe and enabled the NMR analysis of small sample amounts (µg range), usually obtained for natural products. Spectra were analyzed by Bruker TopSpin 3.5 pl 7 and ACDLabs Spectrus Processor. NMR spectra were recorded in DMSO-*d_6_* 99.9 atom%D (Armar Chemicals, Dӧttingen, Switzerland).

HRESIMS data were measured in the positive ion mode on an Orbitrap LQT XL mass spectrometer (Thermo Scientific, Waltham, MA, USA). HPLC-PDA-ELSD-ESIMS data were recorded in positive- and negative-ion mode (scan range of *m/z* 200−1500) on a Shimadzu LC-MS/MS 8030 triple quadrupole MS system (Kyoto, Japan) connected via a T-splitter (1:10) to a photo diode array detector (PDA) (SPD-M20A, Shimadzu, Kyoto, Japan), and evaporative light scattering detector (ELSD) (3300, Alltech, Büchi, Flawil, Switzerland). For data acquisition and processing, LabSolutions software (Kyoto, Japan) was used.

Separations were performed on a C18 SunFire column (3.0 × 150 mm i. d., 3.5 μm) equipped with a precolumn (10 × 3.0 mm i. d.) (Waters). 

Microfractionation was carried out with the same HPLC instrument connected via a T split to an FC204 fraction collector (Gilson, Mettmenstetten, Switzerland) with only UV detection, using a SunFire C_18_ (3.5 μm, 150 × 3.0 mm i.d.) column equipped with a guard column (10 × 3.0 mm i.d.) (Waters). Semipreparative HPLC were performed on an Agilent 1100 system (Santa Clara, CA, USA) with PDA detector. A SunFire C_18_ column (5 μm, 10 × 150 mm i. d.) fitted with a guard column (10 × 10 mm i.d.) (Waters) and a Nucleodur Hilic column (5 μm, 10 × 150 mm i. d.) (Macherey-Nagel), were used for separations. ChemStation software (Agilent Technologies) was used for data acquisition and processing. Preparative HPLC was carried out on a PuriFlash 4100 system (Interchim, Montluçon, France). Separations were performed on Redi*Sep* Rf Gold ^®^-C18 MPLC cartridge 100g (Teledyne Isco).

### 3.2. Plant Material

Roots of *Haplophyllum tuberculatum* were collected in February 2018 in Khartoum, Sudan. The taxonomic identity was confirmed by the Medicinal and Aromatic Plants Research Institute, Sudan. A voucher specimen (HTR 02) is deposited at the Herbarium of the Faculty of Pharmacy, University of Science and Technology, Omdurman, Sudan. The plant material was dried at room temperature and milled with a hammer mill before extraction.

### 3.3. Extraction

The powdered roots of *H. tuberculatum* (300 g) were extracted with 1 L of 70% ethanol under stirring for 24 h. The extract was filtered through Whatman no. 1 filter paper and concentrated in a rotary evaporator to obtain 35.7 g of extract. The extract amount was suspended in water and partitioned consecutively with petroleum ether, chloroform, and ethyl acetate. Three repetitive partitioning procedures, each with 500 mL of either solvent, were performed. In total, 0.4 g of petroleum ether fraction, 4.0 g of chloroform fraction, 2.0 g of ethyl acetate fraction, and 16.4 g of the water fraction were obtained after evaporation. 

### 3.4. Microfractionation for Activity Profiling

HPLC-based microfractionation of the chloroform and the water fractions was performed [H_2_O + 0.1% formic acid (A), CH_3_CN + 0.1% formic acid (B); 0→100% B (0–30 min), 100% B (30–40 min); flow rate 0.4 mL/min; sample concentration 10 mg/mL in DMSO; injection volume 2 × 35 μL] by collecting one-minute fractions from minute 1 to 40 into a 96-deep-well plate. Plates were then dried in a Genevac EZ-2 evaporator (Ipswich, UK) and prepared for antiprotozoal activity testing based on previously established protocols [20,59].

### 3.5. Preparative Isolation

The chloroform fraction was reconstituted in DMSO and separated on a Redi*Sep* Rf Gold ^®^ RP-C18, 100 gm cartridge [H_2_O (A), CH_3_CN (B); 5→100% B (0–120 min), flow rate 20 mL/min]. A total of 13 subfractions (A-M) were combined based on TLC patterns. The subfractions were analyzed by HPLC-PDA-MS to track peaks previously detected in the active time windows of the activity profile.

Subfraction B (247 mg) was submitted to semipreparative HPLC on a C_18_ column [H_2_O (A), CH_3_CN (B); 56% B (0–35 min), 56→100% B (35–40 min), 100% B (40–45 min), flow rate 4 mL/min; sample concentration 50 mg/mL in DMSO; injection volume 50 μL; detection at 208 nm] to afford tetrahydrofuroguaiacin B (**1**, 2.2 mg, t_R_ 22.3 min) and nectandrin B (**2**, 1.9 mg, t_R_ 29.5 min).

A portion (200 mg) of subfraction C was separated by semipreparative HPLC on a C_18_ column [H_2_O (A), MeOH (B); 39→48% B (0–30 min), 48→100% B (30–40 min), 100% B (40–45 min), flow rate 4 mL/min; sample concentration 50 mg/mL in DMSO; injection volume 50 μL; detection at 254 nm], and dihydro-feruloyltyramine (**5**, 2.5 mg, t_R_ 9.4 min), (*E*)-*N*-feruloyltyramine (**6**, 10.6 mg, t_R_ 16.2 min), furoguaiaoxidin (**7**, 1.4 mg, t_R_ 16.2 min), *N*,*N*′-diferuloylputrescine (**8**, 1.4 mg, t_R_ 23.4 min), 7′-ethoxy-trans-feruloyltyramine (**9**, 1.1 mg, t_R_ 27.3 min), and 3,3′-dimethoxy-4,4′-dihydroxylignan-9-ol (**10**, 2.5 mg, t_R_ 36.5 min) were obtained.

For the water fraction, an aliquot (8 g) was re-dissolved in water and separated by preparative HPLC on a Redi*Sep* Rf Gold ^®^ RP-C18, 100 gm cartridge [H_2_O (A), CH_3_CN (B); 20→65% B (0–120 min), 65→100% B (120–135 min), flow flow rate 20 mL/min]. Fractions with similar TLC patterns were combined to yield 15 subfractions (A-O). HPLC-PDA-MS analysis located the peaks detected in the active time window in subfractions **J** and **K**. Subfraction **J** (17 mg) was submitted to semipreparative HPLC on a C18 column (H_2_O (A), CH_3_CN (B); 41% B (0–40 min), 41→100% B (40–45 min), 100% B (45–50 min), flow rate 4 mL/min; sample concentration 50 mg/mL in DMSO; injection volume 50 μL; detection at 193 nm) to afford compounds **11** and **12** as a mixture (2 mg, t_R_ 22.3 min). 

Subfraction K (53 mg) was submitted to semipreparative HPLC on a C18 column (H_2_O (A), CH_3_CN (B); 40% B (0–40 min), 40→100% B (40–45 min), 100% B (45–50 min), flow rate 4 mL/min; sample concentration 50 mg/mL in DMSO; injection volume 50 μL; detection at 193 nm), yielding subfraction K_3_. Subfraction K_3_ (16 mg) was further purified by semipreparative HPLC on a Nucleodur Hilic column (H_2_O (A), CH_3_CN (B); 92% B (0–35 min), 92→20% B (35–45 min), flow rate 4 mL/min; sample concentration 50 mg/mL in DMSO; injection volume 50 μL; detection at 193 nm), to afford compounds **11** and **13** as a mixture (4.2 mg, t_R_ 25.2 min).

*Tetrahydrofuroguaiacin* (**1**): amorphous solid; [α]^25^_D_ −1.9 (*c* 0.2, MeOH); ^1^H and ^13^C NMR, see Table S1, Supporting Information; ESI-MS *m/z* 345 [M + H]^+^. 

*Nectandrin B* (**2**): amorphous solid; [α]^25^_D_ −1.2 (*c* 0.2, MeOH); ^1^H and ^13^C NMR, see Table S1, Supporting Information; ESI-MS *m/z* 345 [M + H]^+^. 

*Fragransin B_2_* (**3**): amorphous solid; ESIMS *m/z* 405 [M + H]^+^.

*Fragransin B_1_* (**4**): amorphous solid; ESIMS *m/z* 405 [M + H]^+^.

*Dihydro-feruloyltyramine* (**5**): amorphous solid; ^1^H and ^13^C NMR, see Table S2, Supporting Information; ESIMS *m/z* 316 [M + H]^+^.

(*E*)-*N*-*Feruloyltyramine* (**6**): amorphous solid; ^1^H and ^13^C NMR, see Table S2, Supporting Information; ESIMS *m/z* 314 [M + H]^+^.

*Furoguaiaoxidin* (**7**): amorphous solid; ^1^H and ^13^C NMR, see Table S2, Supporting Information; ESIMS *m/z* 357 [M + H]^+^.

*N,N′-Diferuloylputrescine* (**8**): amorphous solid; ^1^H and ^13^C NMR, see Table S2, Supporting Information; ESIMS *m/z* 441 [M + H]^+^.

*7′-Ethoxy-trans-feruloyltyramine* (**9**): amorphous solid; [α]^25^_D_ −2.6 (*c* 0.11, MeOH); UV λ_max_ (MeOH) (log ε) 228 (0.23), 288 (0.12), 320 (0.11) nm; ECD (MeOH, *c* 2.0 × 10^−4^ M, 1 mm path length) λ_max_(Δε) 207 (+4.04), 224 (+2.40), 235 (+1.72) nm; ^1^H and ^13^C NMR, see Table S3, Supporting Information; ESIMS *m/z* 358 [M + H]^+^.

*3,3′-Dimethoxy-4,4′-dihydroxylignan-9-ol* (**10**): amorphous solid; [α]^25^_D_ −4.9 (*c* 0.25, MeOH); UV λ_max_ (MeOH) (log ε) 230 (0.18), 280 (0.08) nm; ECD (MeOH, *c* 1.4 × 10^−4^ M, 1 mm path length) λ_max_(Δε) 205 (+3.07), 215 (+1.39), 235 (+0.89) nm; ^1^H and ^13^C NMR, see Table S3, Supporting Information; ESIMS *m/z* 693 [2M + H]^+^.

(3*S*,20*S*,22*R*,25*S*)-Spirost-5-en-3-yl (β-d-xylopyranosyl-(1→3)- β-d-glucopyranosyl-(1→4)[α-l- rhamnopyranosyl-(1→2)]-β-d-glucopyranoside (**11**): amorphous solid; ^1^H and ^13^C NMR, see Table S4, Supporting Information; HRESIMS *m*/*z* 1039.5083 [M + Na]^+^ (calcd for C_50_H_80_O_21_Na^+^ 1039.5085). 

(3*S*,20*S*,22*R*,25*S*)-Spirost-5-en-3-yl (β-d-rhamnopyranosyl-(1→3)- β-d-glucopyranosyl-(1→4)[α-l- rhamnopyranosyl-(1→2)]-β-d-glucopyranoside (**12**): amorphous solid; ^1^H and ^13^C NMR, see Table S4, Supporting Information; HRESIMS *m*/*z* 1053.5233 [M + Na]^+^ (calcd for C_51_H_82_O_21_Na^+^ 1053.5241). 

(3*S*,20*S*,22*R*,25*R*)-Spirost-5-en-3-yl (β-d-xylopyranosyl-(1→3)-β-d-glucopyranosyl-(1→4)[α-l- rhamnopyranosyl-(1→2)]-β-d-glucopyranoside (**13**)**:** amorphous solid; ^1^H and ^13^C NMR, see Table S4, Supporting Information; HRESIMS *m*/*z* 1039.5072 [M + Na]^+^ (calcd for C_50_H_80_O_21_Na^+^ 1039.5085).

### 3.6. Activity against Leishmania Donovani Axenic Amastigotes

Amastigotes of *L. donovani* strain MHOM/ET/67/L82 were grown under an atmosphere of 5% CO_2_ in air in axenic culture at 37 °C in SM medium [60] at pH 5.4 supplemented with 10% heat-inactivated fetal bovine serum. Next, 50 µL of culture medium was added in the wells of a 96-well plate and serial drug dilutions of eleven 3-fold dilution steps covering a final range from 100 to 0.002 μg/mL were prepared. Then, 50 µL of culture medium with 2 × 10^5^ amastigotes from axenic culture were added to each well. The plates were incubated for 70 h and then inspected under an inverted microscope to assure growth of the controls and sterile conditions. 10 μL of resazurin (12.5 mg resazurin dissolved in 100 mL distilled water) were added to each well of the plates and allowed for additional 2 h incubation. Afterwards, plates were read with a Spectramax Gemini XS microplate fluorometer (Molecular Devices Cooperation, Sunnyvale, CA, USA) using an excitation wavelength of 536 nm and an emission wavelength of 588 nm. Data were analyzed using the software Softmax Pro (Molecular Devices Cooperation, Sunnyvale, CA, USA). Decrease of fluorescence (=inhibition) was expressed as percentage of the fluorescence of untreated control cultures and plotted against the drug concentrations. From the sigmoidal inhibition curves the IC_50_ values were calculated by linear regression using Microsoft Excel. Miltefosine was used as positive control drug. Assays were performed in two independent replicates at least.

### 3.7. Activity against Leishmania Donovani Intramacrophage Amastigotes

Mouse peritoneal macrophages (4 × 10^4^ in 100 µL RPMI 1640 medium with 10% heat-inactivated FBS) were seeded into wells of a 96-well plate. After 24 h, 100 µL of 2 × 10^5^ amastigote *Leishmania donovani* were added. The amastigotes were taken from an axenic amastigote culture grown at pH 5.4. The medium containing free amastigote forms was removed after 24 h and replaced with fresh medium. The washing step was repeated and afterwards the serial drug dilution was prepared with at least 6 dilution steps. Compound was dissolved in DMSO at 10 mg/mL and further diluted in medium. After incubation for 96 h at 37 °C under a 5% CO_2_ atmosphere, the medium was removed and cells were fixed by adding 50 µL 4% formaldehyde solution followed by a staining with a 5 µM DRAQ5 solution. Plates were imaged in ImageXpress XLS (MD) microscope using a 20× air objective (635 nm excitation: 690/50 emission). Nine images were collected per well. Automated image analysis was performed with a script developed on Meta Xpress Software (MD). Three outputs were provided for each well: (i) number of host cell nuclei; (ii) numbers of infected and non-infected host cells; (iii) number of parasite nuclei per infected host cell. The IC_50_ values were calculated based on the infection rate and the numbers of intracellular amastigotes. Miltefosine was used as control. Assays were performed in two independent replicates at least.

### 3.8. Activity against Plasmodium Falciparum

In vitro activity against the erythrocytic stages of *P. falciparum* was determined using a ^3^H-hypoxanthine incorporation assay [61], using the drug-sensitive NF54 strain [62]. Compounds were dissolved in DMSO at 10 mg/mL and further diluted in medium before addition to parasite cultures incubated in RPMI 1640 medium without hypoxanthine, supplemented with HEPES (5.94 g/L), NaHCO_3_ (2.1 g/L), neomycin (100 U/mL), Albumax^R^ (5 g/L) and washed human red cells A^+^ at 2.5% haematocrit (0.3% parasitaemia). Serial drug dilutions of eleven 3-fold dilution steps covering a range from 100 to 0.002 μg/mL were prepared. The 96-well plates were incubated in a humidified atmosphere at 37 °C; 4% CO_2_, 3% O_2_, 93% N_2_. After 48 h 50 μL of ^3^H-hypoxanthine (=0.5 μCi) was added to each well of the plate. The plates were incubated for a further 24 h under the same conditions. The plates were then harvested with a Betaplate™ cell harvester (Wallac, Zurich, Switzerland), and the red blood cells transferred onto a glass fibre filter then lysed with distilled water. The dried filters were inserted into a plastic foil with 10 mL of scintillation fluid, and counted in a Betaplate™ liquid scintillation counter (Wallac, Zurich, Switzerland). IC_50_ values were calculated from sigmoidal inhibition curves by linear regression using Microsoft Excel. Chloroquine (Sigma C6628) was used as positive control. Assays were performed in two independent replicates at least.

### 3.9. Activity against Trypanosoma Brucei Rhodesiense

The stock was originally isolated from a Tanzanian patient and adapted to axenic culture conditions after several mouse passages and cloned. Minimum Essential Medium (50 µL) supplemented with 25 mM HEPES, 1 g/L additional glucose, 1% MEM non-essential amino acids (100×), 0.2 mM 2-mercaptoethanol, 1mM Na-pyruvate [63] and 15% heat inactivated horse serum was added to each well of a 96-well microtiter plate. Serial drug dilutions of eleven 3-fold dilution steps covering a range from 100 to 0.002 μg/mL were prepared. Then 4 × 10^3^ bloodstream forms of *T. b. rhodesiense* STIB 900 in 50 µL were added to each well and the plate incubated at 37 °C under a 5% CO_2_ atmosphere for 70 h. 10 µL resazurin solution (resazurin, 12.5 mg in 100 mL double-distilled water) was then added to each well and incubation continued for a further 2–4 h [64]. Then the plates were read with a Spectramax Gemini XS microplate fluorometer (Molecular Devices Cooperation, Sunnyvale, CA, USA) using an excitation wavelength of 536 nm and an emission wavelength of 588 nm. Data were analyzed with the graphic programme Softmax Pro (Molecular Devices Cooperation, Sunnyvale, CA, USA), which calculated IC_50_ values by linear regression [65], and 4-parameter logistic regression from the sigmoidal dose inhibition curves using Microsoft Excel. Melarsoprol (Arsobal Sanofi-Aventis, received from WHO) was used as control. Assays were performed in two independent replicates at least.

### 3.10. In Vitro Cytotoxicity with L-6 Cells

Assays were performed in 96-well microtiter plates, each well containing 100 μL of RPMI 1640 medium supplemented with 1% l-glutamine (200 mM) and 10% fetal bovine serum, and 4000 L-6 cells (a primary cell line derived from rat skeletal myoblasts) [66]. Serial drug dilutions of eleven 3-fold dilution steps covering a range from 100 to 0.002 μg/mL were prepared 24 h post seeding L-6 cells. After 70 h of incubation the plates were inspected under an inverted microscope to assure growth of the controls and sterile conditions. 10 μL of resazurin was then added to each well and the plates incubated for another 2 h. Then the plates were read with a Spectramax Gemini XS microplate fluorometer (Molecular Devices Cooperation, Sunnyvale, CA, USA) using an excitation wavelength of 536 nm and an emission wavelength of 588 nm. The IC_50_ values were calculated by linear regression and 4-parameter logistic regression from the sigmoidal dose inhibition curves using SoftmaxPro software (Molecular Devices Cooperation, Sunnyvale, CA, USA) and Microsoft Excel. Podophyllotoxin (Sigma P4405) was used as control. All assays were performed in two independent replicates at least.

## 4. Conclusions

In total, eleven compounds (**1**, **2**, and **5**–**13)** have been reported here for the first time from *Haplophyllum* species and the Rutaceae family. It is interesting to note that, to the best of our knowledge, this is the first report on the presence of the saponin class in the respective plant species and family. The antiprotozoal activity of these compounds, plus the two stereoisomeric tetrahydrofuran lignans fragransin B_2_ (**3**) and fragransin B_1_ (**4**), was determined against *L. donovani* amastigotes, *P. falciparum,* and *T. b. rhodesiense* bloodstream forms, along with their cytotoxicity to rat myoblast L6 cells. Nectandrin B (**2**) exhibited the highest activity against *L. donovani* axenic amastigotes (IC_50_ 4.5 µM) and the highest selectivity index (SI 25.5). The lignan 3,3′-dimethoxy-4,4′-dihydroxylignan-9-ol (**10**) was the most active against *P. falciparum* (IC_50_ 9.3 µM; SI 13.7). The steroidal saponins (**11**–**13**) showed activity with IC_50_ ˂ 8 µg/mL across all parasites; however, they were the least selective (SI ˂ 2). The promising activity of nectandrin B against axenic *L. donovani* amastigotes was not reproduced when tested against intracellular amastigotes, possibly due to lack of penetration or stability of nectandrin B in macrophages. Nevertheless, the specific antileishmanial activity of nectandrin B could serve as the starting point for the identification of a new antileishmanial drug target.

## Figures and Tables

**Table 1:** In vitro Activity of compounds **1**−**10** against *L. donovani* (MHOM-ET-67/L82) axenic amastigotes, *P. falciparum* (NF54), *T. b. rhodesiense* (STIB 900), and cytotoxicity in L6 Cells.

Compound	*L. donovani*		*P. falciparum*		*T. b. rhodesiense*		L6 Cells
No.	IC_50_ *^a^* (µM)	SI *^b^*	IC_50_ *^a^* (µM)	SI *^b^*	IC_50_ *^a^* (µM)	SI *^b^*	
**1**	22.6 ± 6.5	4.2	53.1 ± 2.0	1.8	59.4 ± 2.5	1.6	95.2 ± 9.3
**2**	4.5 ± 1.0	25.5	9.5 ± 0.1	12.1	48.3 ± 1.3	2.4	115.0 ± 2.6
**3**	36.0 ± 7.3	4.7	36.1 ± 1.0	4.7	47.5 ± 4.5	3.6	2001 ± 47.0
**4**	29.2 ± 6.0	4.8	17.4 ± 2.1	8.0	40.7 ± 0.4	3.4	163.0 ± 4.5
**5**	141.3 ± 1.9	n.d	158.7	n.d	206.0 ± 66.7	n.d	˃317.5
**6**	99.4 ± 9.3	2.5	68.4 ± 3.8	3.6	120.9 ± 36.9	2.0	246.8 ± 3.7
**7**	136.5 ± 1.5	n.d	48.1 ± 10.9	n.d	114.0 ± 0.0	n.d	˃280.9
**8**	97.0 ± 0.9	1.5	30.6 ± 8.3	4.8	18.9 ± 8.4	7.8	146.5 ± 18.8
**9**	69.6 ± 4.6	3.0	30.4 ± 8.0	6.8	72.7 ± 30.4	2.9	207.8 ± 21.6
**10**	55.3 ± 3.0	2.3	9.3 ± 1.3	13.7	27.5 ± 8.4	4.6	127.6 ± 3.9
Positive control	0.5 *^d^*		0.01 *^e^*		0.01 *^c^*		0.03 *^f^*

*^a^* The IC_50_s are mean values from at least two independent replicates ± absolute deviation. *^b^* Selectivity index (SI): IC_50_ in L6 cells divided by IC_50_ in the titled parasitic strain; *^c^* melarsoprol; *^d^* miltefosine; *^e^* chloroquine; *^f^* podophyllotoxin; n.d: not determined.

## Supplementary Materials

The following are available online at https://www.mdpi.com/1420-3049/25/12/2825/s1, Figure S1: UV (**A**) and ECD (**B**) spectra for compound **9** in MeOH (0.07 mg/mL), Figure S2: UV (**A**) and ECD (**B**) spectra for compound **10** in MeOH (0.05 mg/mL), Figure S3: HSQC-DEPT spectrum of the mixture of compounds **11** (in red and black) and **12** (in blue and black) (500 MHz, DMSO), Figure S4: HMBC spectrum of the mixture of compounds **11** and **12** (500 MHz, DMSO), Figure S5: HSQC-DEPT spectrum of the mixture of compounds **11** and **13** (labelled in red, a prime was added for signals differing from **11**) (500 MHz, DMSO), Figure S6: HMBC spectrum of the mixture of compounds **11** and **13** (500 MHz, DMSO), Table S1: ^1^H and ^13^C NMR Spectroscopic Data for Compounds **1** and **2** (DMSO-*d6*; 500.13 MHz for ^1^H and 125.77 for ^13^C NMR; δ in ppm), Table S2: ^1^H and ^13^C NMR Spectroscopic Data for Compounds **5**–**8** (DMSO-*d6*; 500.13 MHz for ^1^H and 125.77 for ^13^C NMR; δ in ppm), Table S3: ^1^H and ^13^C NMR Spectroscopic Data for Compounds **9** and **10** (DMSO-*d6*; 500.13 MHz for ^1^H and 125.77 for ^13^C NMR; δ in ppm), Table S4: ^1^H and ^13^C NMR Spectroscopic Data for Compounds **11**–**13** (DMSO-*d6*; 600.18 MHz for ^1^H and 125.77 for ^13^C NMR; δ in ppm).
