# Supplementary material for: Lignans, Amides, and Saponins from Haplophyllum tuberculatum and Their Antiprotozoal Activity
Source: Molecules. 2020 Jun 19;25(12):2825. doi: 10.3390/molecules25122825 (PMC7355546; doi:10.3390/molecules25122825)
Supplement: Supplementary file 1 [file molecules-25-02825-s001.pdf]

**SUPPORTING INFORMATION:**

# Lignans, Amides, and Saponins from *Haplophyllum tuberculatum* and their Antiprotozoal Activity

**Abdelhalim Babiker Mahmoud [1,2,3,*], Ombeline Danton [2], Marcel Kaiser [1], Sohee Han [4], Aitor Moreno [5], Shereen Abd Algaffar [6], Sami Khalid [3,6], Won Keun Oh [4], Matthias Hamburger [2] and Pascal Mäser [1,2]**

[1] Swiss Tropical and Public Health Institute, Basel, Switzerland; halim.mahmoud@swisstph.ch; marcel.kaiser@swisstph.ch; pascal.mäser@swisstph.ch
[2] University of Basel, Basel, Switzerland; ombeline.danton@unibas.ch; matthias.hamburger@unibas.ch
[3] Faculty of Pharmacy, University of Khartoum, Khartoum, Sudan; khalidseek@hotmail.com
[4] Korea Bioactive Natural Material Bank, College of Pharmacy, Seoul National University, Seoul 08826, Republic of Korea; wkoh1@snu.ac.kr; sohee.hn@snu.ac.kr
[5] Bruker BioSpin, Fällanden, Switzerland; Aitor.Moreno@bruker.com
[6] Faculty of Pharmacy, University of Science and Technology, Omdurman, Sudan; phd_sh086@hotmail.com
* Correspondence: (A.B.M); halim.mahmoud@swisstph.ch; (P.M); pascal.mäser@swisstph.ch

# Table of Contents

**Table S1.** $^{1}$H and $^{13}$C NMR Spectroscopic Data for Compounds **1** and **2** (DMSO-*d6*; 500.13 MHz for $^{1}$H and 125.77 for $^{13}$C NMR; δ in ppm) .................................................................. **1**

**Table S2.** $^{1}$H and $^{13}$C NMR Spectroscopic Data for Compounds **5-8** (DMSO-*d6*; 500.13 MHz for $^{1}$H and 125.77 for $^{13}$C NMR; δ in ppm) ........................................................................... **2**

**Table S3.** $^{1}$H and $^{13}$C NMR Spectroscopic Data for Compounds **9** and **10** (DMSO-*d6*; 500.13 MHz for $^{1}$H and 125.77 for $^{13}$C NMR; δ in ppm) .................................................................. **3**

**Table S4.** $^{1}$H and $^{13}$C NMR Spectroscopic Data for Compounds **11-13** (DMSO-*d6*; 600.18 MHz for $^{1}$H and 125.77 for $^{13}$C NMR; δ in ppm) .................................................................. **4**

**Figure S1.** UV (**A**) and ECD (**B**) spectra for compound **9** in MeOH (0.07 mg/mL) ............... **6**

**Figure S1.** UV (**A**) and ECD (**B**) spectra for compound **10** in MeOH (0.05 mg/mL) ............. **7**

**Figure S3.** HSQC-DEPT spectrum of the mixture of compounds **11** (in red and black) and **12** (in blue and black) (500 MHz, DMSO)…………………………………………………………..**8**

**Figure S4.** HMBC spectrum of the mixture of compounds **11** and **12** (500 MHz, DMSO)…..**9**

**Figure S5.** HSQC-DEPT spectrum of the mixture of compounds **11** and **13** (labelled in red, a prime was added for signals differing from **11**) (500 MHz, DMSO)………………………...**10**

**Figure S6.** HMBC spectrum of the mixture of compounds **11** and **13** (500 MHz, DMSO)…**11**

**Table S 1.** $^{1}H$ and $^{13}C$ NMR Spectroscopic Data for Compound **1** and **2** (DMSO-*d6*; 500.13 MHz for $^{1}H$ and 125.77 for $^{13}C$ NMR; δ in ppm)

| | **1** | | **2** | |
|---|---|---|---|---|
| Position | $\delta_C$[a] | $\delta_H$ (mult *J* in Hz) | $\delta_C$[a] | $\delta_H$ (mult *J* in Hz) |
| 2 | 81.9, CH | 4.98, d (6.4) | 86.5, CH | 4.34, d (5.8) |
| 3 | 40.7, CH | 2.62, m | 43.8, CH | 2.20, m |
| 4 | 40.7, CH | 2.62, m | 43.8, CH | 2.20, m |
| 5 | 81.9, CH | 4.98, d (6.4) | 86.5, CH | 4.34, d (5.8) |
| 1', 1" | 131.4, C | | 133.1, C | |
| 2', 2" | 110.7, CH | 6.94, s | 110.8, CH | 6.95, s |
| 3', 3" | 147.2, C | | 147.5, C | |
| 4', 4" | 145.3, C | | 146.1, C | |
| 5', 5" | 115.1, CH | 6.79 [b] | 115.3, CH | 6.78[b] |
| 6', 6" | 118.7, CH | 6.79 [b] | 118.8, CH | 6.81[b] |
| 3-Me | 11.5, $CH_3$ | 0.51, d (6.4) | 12.6, $CH_3$ | 0.94, d (6.1) |
| 4-Me | 11.5, $CH_3$ | 0.51, d (6.4) | 12.6, $CH_3$ | 0.94, d (6.1) |
| 3'-OMe, 3"-OMe | 55.6, $CH_3$ | 3.76, s | 55.6, $CH_3$ | 3.75, s |

[a] $^{13}C$ NMR data extracted from HSQC and HMBC spectra, [b] Overlapping signals.

**Table S2.** $^1$H and $^{13}$C NMR Spectroscopic Data for Compounds **5-8** (DMSO-*d6*; 500.13 MHz for $^1$H and 125.77 for $^{13}$C NMR; $\delta$ in ppm)

| | **5** | | **6** | | **7** | | **8** | |
|---|---|---|---|---|---|---|---|---|
| Position | $\delta_C$[a] | $\delta_H$ (mult *J* in Hz) | $\delta_C$[a] | $\delta_H$ (mult *J* in Hz) | $\delta_C$[a] | $\delta_H$ (mult *J* in Hz) | $\delta_C$[a] | $\delta_H$ (mult *J* in Hz) |
| 1 | 132.2, C | | 126.8, C | | [c] | | [c] | |
| 2 | 112.7, CH | 6.76, d (1.5) | 111.3, CH | 7.14, br s | 112.0, CH | 7.01, d (1.5) | 111.0, CH | 7.11, br s |
| 3 | 147.4, C | | 148.1, C | | [c] | | [c] | |
| 4 | 144.8, C | | 148.6, C | | [c] | | [c] | |
| 5 | 115.2, CH | 6.70 [b] | 116.1, CH | 6.87, d (8.2) | 114.8, CH | 6.76, d (8.2) | 115.9, CH | 6.82, d (8.2) |
| 6 | 120.3, CH | 6.59, dd (7.8, 1.4) | 121.9, CH | 7.03 [b] | 124.5, CH | 7.14, dd (8.2, 1.5) | 121.5, CH | 6.98, br d (8.2) |
| 7 | 30.8, $CH_2$ | 2.72, t (7.6) | 139.6, CH | 7.46, d (15.6) | [c] | | 138.8, CH | 7.32, d (15.9) |
| 8 | 37.4, $CH_2$ | 2.34, t (7.8) | 119.3, CH | 6.56, d (15.6) | [c] | | 119.3, CH | 6.46, d (15.9) |
| 9 | 171.6, C | | 166.2, C | | 16.9, $CH_3$ | 2.03, s | [c] | |
| 10 | | | | | | | 38.3, $CH_2$ | 3.20 [b] |
| 11 | | | | | | | 26.9, $CH_2$ | 1.50, m |
| 1' | 132.2, C | | 129.8, C | | [c] | | [c] | |
| 2' | 129.3, CH | 6.95, d (8.4) | 129.7, CH | 7.05 [b] | 112.0, CH | 7.01, d (1.5) | 111.0, CH | 7.11, br s |
| 3' | 115.2, CH | 6.70 [b] | 115.5, CH | 6.76, d (8.2) | [c] | | [c] | |
| 4' | 155.7, C | | 155.9, C | | [c] | | [c] | |
| 5' | 115.2, CH | 6.70 [b] | 115.5, CH | 6.76, d (8.2) | 114.8, CH | 6.76, d (8.2) | 115.9, CH | 6.82, d (8.2) |
| 6' | 129.3, CH | 6.95, d (8.4) | 129.7, CH | 7.05 [b] | 124.5, CH | 7.14, dd (8.2, 1.5) | 121.5, CH | 6.98, br d (8.2) |
| 7' | 34.4, $CH_2$ | 2.58, t (7.3) | 34.7, $CH_2$ | 2.73, t (7.0) | [c] | | 138.8, CH | 7.32, d (15.9) |
| 8' | 40.5, $CH_2$ | 3.22, dt (5.2, 6.7) | 40.9, $CH_2$ | 3.45, dt (5.5, 5.5) | [c] | | 119.3, CH | 6.46, d (15.9) |
| 9' | | | | | 16.9, $CH_3$ | 2.03, s | [c] | |
| 10' | | | | | | | 38.3, $CH_2$ | 3.20 [b] |
| 11' | | | | | | | 26.9, $CH_2$ | 1.50, m |
| 3-OMe | 55.7, $CH_3$ | 3.75, s | 55.9, $CH_3$ | 3.81, s | | | | |
| 3'-OMe | | | | | 55.5, $CH_3$ | 3.70, s | 55.5, $CH_3$ | 3.81, s |
| NH | | 7.77, t (5.2) | | 7.97, t (5.5) | | | | 7.93, t (4.9) |

[a] $^{13}$C NMR data extracted from HSQC and HMBC spectra, [b] Overlapping signals, [c] Signal not visible due to low amount of compound.

**Table S3.** $^{1}H$ and $^{13}C$ NMR Spectroscopic Data for Compounds **9-10** (DMSO-*d6*; 500.13 MHz for $^{1}H$ and 125.77 for $^{13}C$ NMR; δ in ppm)

| | **9** | | **10** | |
|---|---|---|---|---|
| Position | $\delta_C$[a] | $\delta_H$ (mult *J* in Hz) | $\delta_C$[a] | $\delta_H$ (mult *J* in Hz) |
| 1 | 126.3, C | | 132.3, C | |
| 2 | 111.1, CH | 7.11 [b] | 113.1, CH | 6.68 [b] |
| 3 | 147.8, C | | 147.2, C | |
| 4 | 147.6, C | | 144.2, C | |
| 5 | 115.7, CH | 6.82, d (7.9) | 115.0, CH | 6.70 [b] |
| 6 | 121.7, CH | 6.97, dd (7.9, 1.5) | 121.0, CH | 6.57, dt (7.8, 2.5) |
| 7 | 139.1, CH | 7.31, d (15.6) | 32.0, $CH_2$ | 2.56, dd (13.7, 5.5)<br>2.32 [b] |
| 8 | 119.0, CH | 6.52, d (15.9) | 45.7, CH | 1.67, m |
| 9 | 165.5, C | | 61.0, $CH_2$ | 3.34, dd (12.5, 7.0) |
| 1' | 130.5, C | | 132.3, C | |
| 2' | 127.7, CH | 7.11 [b] | 113.1, CH | 6.68 [b] |
| 3' | 115.1, CH | 6.77, d (8.2) | 147.2, C | |
| 4' | 157.2, C | | 144.2, C | |
| 5' | 115.1, CH | 6.77, d (8.2) | 115.0, CH | 6.70 [b] |
| 6' | 127.7, CH | 7.11 [b] | 121.0, CH | 6.57, dt (7.8, 2.5, 2.5) |
| 7' | 79.5, CH | 4.28, dd (7.5, 5.0) | 39.1, $CH_2$ | 2.64, dd (13.7, 6.4)<br>2.32 [b] |
| 8' | 45.3, $CH_2$ | 3.34, d | 33.6, CH | 2.04, m |
| 9' | | | 15.1, $CH_3$ | 0.82, d (6.7) |
| 1" | 63.3, $CH_2$ | 3.31, q (7.0) | | |
| 2" | 15.1, $CH_3$ | 1.10, t (6.9) | | |
| 3-OMe | 55.7, $CH_3$ | 3.81, s | 55.4, $CH_3$ | 3.73, s |
| 3'-OMe | | | 55.4, $CH_3$ | 3.73, s |
| NH | | 7.86, t (5.5) | | |

[a] $^{13}C$ NMR data extracted from HSQC and HMBC spectra, [b] Overlapping signals.

**Table S4.** $^{1}$H and $^{13}$C NMR Spectroscopic Data for Compounds **11-13** (DMSO-*d6*; 600.18 Hz for $^{1}$H and 150.92 for $^{13}$C NMR; δ in ppm)

| | 11 | | 12 | | 13 | |
|---|---|---|---|---|---|---|
| Position | $\delta_C{}^a$ | $\delta_H$ (mult *J* in Hz) | $\delta_C{}^a$ | $\delta_H$ (mult *J* in Hz) | $\delta_C{}^a$ | $\delta_H$ (mult *J* in Hz) |
| 1 | 37.2, $CH_2$ | 1.78 $^b$<br>0.96 $^b$ | 37.2, $CH_2$ | 1.78 $^b$<br>0.96 $^b$ | 37.1, $CH_2$ | 1.76 $^b$<br>0.96 $^b$ |
| 2 | 29.4, $CH_2$ | 1.77 $^b$<br>1.44 $^b$ | 29.4, $CH_2$ | 1.77 $^b$<br>1.44 $^b$ | 29.4, $CH_2$ | 1.78 $^b$<br>1.43 $^b$ |
| 3 | 76.9, CH | 3.46 $^b$ | 76.9, CH | 3.46 $^b$ | 76.9, CH | 3.46 $^b$ |
| 4 | 38.1, $CH_2$ | 2.39, br d (11.4)<br>2.15, dd (11.4, 11.4) | 38.1, $CH_2$ | 2.39, br d (11.4)<br>2.15, dd (11.4, 11.4) | 38.0, $CH_2$ | 2.38 $^c$<br>2.15 $^c$ |
| 5 | 140.7, C | | 140.7, C | | 140.6, C | |
| 6 | 121.8, CH | 5.33, m | 121.8, CH | 5.33, m | 121.5, CH | 5.30 $^c$ |
| 7 | 31.9, $CH_2$ | 1.91 $^b$<br>1.49 $^b$ | 31.9, $CH_2$ | 1.91 $^b$<br>1.49 $^b$ | 31.8, $CH_2$ | 1.92 $^b$<br>1.47 $^b$ |
| 8 | 31.4, CH | 1.54 $^b$ | 31.4, CH | 1.54 $^b$ | 31.3, CH | 1.53 $^b$ |
| 9 | 50.0, CH | 0.88 $^b$ | 50.0, CH | 0.88 $^b$ | 50.0, CH | 0.87 $^b$ |
| 10 | 36.8, C | | 36.8, C | | 36.8, C | |
| 11 | 20.8, $CH_2$ | 1.47 $^b$<br>1.38 $^b$ | 20.8, $CH_2$ | 1.47 $^b$<br>1.38 $^b$ | 20.7, $CH_2$ | 1.46 $^b$<br>1.37 $^b$ |
| 12 | 39.5, $CH_2$ | 1.67 $^b$<br>1.12 $^b$ | 39.5, $CH_2$ | 1.67 $^b$<br>1.12 $^b$ | 39.5, $CH_2$ | 1.67 $^b$<br>1.11 $^b$ |
| 13 | 40.2, C | | 40.2, C | | 39.8, C | |
| 14 | 56.2, CH | 1.07 $^b$ | 56.2, CH | 1.07 $^b$ | 56.2, CH | 1.05 $^b$ |
| 15 | 31.8, $CH_2$ | 1.88 $^b$<br>1.17 $^b$ | 31.8, $CH_2$ | 1.88 $^b$<br>1.17 $^b$ | 31.4, $CH_2$ | 1.86 $^b$<br>1.17 $^b$ |
| 16 | 80.7, CH | 4.27 $^b$ | 80.7, CH | 4.27 $^b$ | 80.5, CH | 4.26 $^b$ |
| 17 | 62.0, CH | 1.65 $^b$ | 62.0, CH | 1.65 $^b$ | 61.8, CH | 1.64 $^b$ |
| 18 | 16.4, $CH_3$ | 0.72, s | 16.4, $CH_3$ | 0.72, s | 16.3, $CH_3$ | 0.71 $^b$ |
| 19 | 19.4, $CH_3$ | 0.94, s | 19.4, $CH_3$ | 0.94, s | 19.2, $CH_3$ | 0.93, s |
| 20 | 42.0, CH | 1.75 $^b$ | 42.0, CH | 1.75 $^b$ | 41.4, CH | 1.78 $^b$ |
| 21 | 14.9, $CH_3$ | 0.92, d (5.6) | 14.9, $CH_3$ | 0.92, d (5.6) | 14.6, $CH_3$ | 0.88 $^c$ |
| 22 | 109.4, C | | 109.4, C | | 108.5, C | |
| 23 | 25.9, $CH_2$ | 1.80 $^b$<br>1.26 $^b$ | 25.9, $CH_2$ | 1.80 $^b$<br>1.26 $^b$ | 30.9, $CH_2$ | 1.60 $^b$<br>1.45 $^b$ |
| 24 | 25.8, $CH_2$ | 1.87 $^b$<br>1.33 $^b$ | 25.8, $CH_2$ | 1.87 $^b$<br>1.33 $^b$ | 28.5, $CH_2$ | 1.54 $^b$<br>1.29 $^b$ |
| 25 | 26.8, CH | 1.63 $^b$ | 26.8, CH | 1.63 $^b$ | 30.1, CH | 1.50 $^b$ |
| 26 | 64.7, $CH_2$ | 3.78, br d (10.0)<br>3.21 $^b$ | 64.7, $CH_2$ | 3.78, br d (10.0)<br>3.21 $^b$ | 65.9, $CH_2$ | 3.38 $^b$<br>3.17 $^b$ |
| 27 | 16.3, $CH_3$ | 0.99, d (6.0) | 16.3, $CH_3$ | 0.99, d (6.0) | 17.4, $CH_3$ | 0.71 $^b$ |
| 1- Glc1 | 98.4, CH | 4.43, d (7.5) | 98.4, CH | 4.43, d (7.5) | 98.4, CH | 4.41 $^b$ |
| 2- Glc1 | 76.4, CH | 3.22 $^b$ | 76.4, CH | 3.22 $^b$ | 76.0, CH | 3.23 $^b$ |
| 3- Glc1 | 76.4, CH | 3.50 $^b$ | 76.4, CH | 3.50 $^b$ | 76.3, CH | 3.51 $^b$ |
| 4- Glc1 | 81.1, CH | 3.32 $^b$ | 81.1, CH | 3.32 $^b$ | 81.0, CH | 3.34 $^b$ |
| 5- Glc1 | 74.9, CH | 3.28 $^b$ | 74.9, CH | 3.28 $^b$ | 74.7, CH | 3.27 $^b$ |
| 6- Glc1 | 60.5, $CH_2$ | 3.69 $^b$<br>3.58 $^b$ | 60.5, $CH_2$ | 3.69 $^b$<br>3.58 $^b$ | 60.5, $CH_2$ | 3.70 $^b$<br>3.59 $^b$ |

$^a$ $^{13}$C NMR data extracted from HSQC and HMBC spectra, $^b$ Overlapping signals, $^c$ broad signal due to concentrated sample.

(continued next page)

**Table S4.** (continued)

| | 11 | | 12 | | 13 | |
|---|---|---|---|---|---|---|
| Position | $\delta_C$[a] | $\delta_H$ (mult *J* in Hz) | $\delta_C$[a] | $\delta_H$ (mult *J* in Hz) | $\delta_C$[a] | $\delta_H$ (mult *J* in Hz) |
| 1- Rha1 | 100.4, CH | 5.04, br s | 100.4, CH | 5.04, br s | | |
| 2- Rha1 | 70.8, CH | 3.63 [b] | 70.8, CH | 3.63 [b] | 70.7, CH | 3.64 [b] |
| 3- Rha1 | 71.0, CH | 3.40 [b] | 71.0, CH | 3.40 [b] | 70.9, CH | 3.40 [b] |
| 4- Rha1 | 72.3, CH | 3.18 [b] | 72.3, CH | 3.18 [b] | 72.2, CH | 3.19 [b] |
| 5- Rha1 | 68.3, CH | 3.97, m | 68.3, CH | 3.97, m | 68.2, CH | 3.97 [c] |
| 6- Rha1 | 18.2, $CH_3$ | 1.07 [b] | 18.2, $CH_3$ | 1.07 [b] | 18.0, $CH_3$ | 1.08 [b] |
| 1- Glc2 | 103.0, CH | 4.34, d (7.5) | 103.2, CH | 4.28 [b] | 102.9, CH | 4.34 [b] |
| 2- Glc2 | 73.1, CH | 3.18 [b] | 74.4, CH | 3.09 [b] | 72.9, CH | 3.20 [b] |
| 3- Glc2 | 86.4, CH | 3.40 [b] | 81.3, CH | 3.38 [b] | 86.2, CH | 3.41 [b] |
| 4- Glc2 | 68.4, CH | 3.16 [b] | 68.7, CH | 3.11 [b] | 68.3, CH | 3.16 [b] |
| 5- Glc2 | 76.9, CH | 3.24 [b] | 77.0, CH | 3.24 [b] | 76.4, CH | 3.26 [b] |
| 6- Glc2 | 61.1, $CH_2$ | 3.66 [b]<br>3.42 [b] | 61.1, $CH_2$ | 3.66 [b]<br>3.42 [b] | 60.9, $CH_2$ | 3.68 [b]<br>3.41 [b] |
| 1- Xyl | 105.0, CH | 4.38, d (7.1) | - | - | 104.7, CH | 4.38 [b] |
| 2- Xyl | 74.1, CH | 3.05 [b] | - | - | 73.9, CH | 3.06 [b] |
| 3- Xyl | 76.6, CH | 3.13 [b] | - | - | 76.1, CH | 3.14 [b] |
| 4- Xyl | 69.8, CH | 3.29 [b] | - | - | 69.6, CH | 3.30 [b] |
| 5- Xyl | 66.2, $CH_2$ | 3.72 [b]<br>3.06 [b] | - | - | 66.0, $CH_2$ | 3.73 [b]<br>3.08 [b] |
| 1- Rha2 | - | - | 101.0, CH | 5.01, br s | | |
| 2- Rha2 | - | - | 70.9, CH | 3.69 [b] | | |
| 3- Rha2 | - | - | 70.9, CH | 3.47 [b] | | |
| 4- Rha2 | - | - | 72.4, CH | 3.17 [b] | | |
| 5- Rha2 | - | - | 68.5, CH | 3.87, m | | |
| 6- Rha2 | - | - | 18.2, $CH_3$ | 1.07 [b] | | |

[a] $^{13}C$ NMR data extracted from HSQC and HMBC spectra, [b] Overlapping signals, [c] broad signal due to concentrated sample.

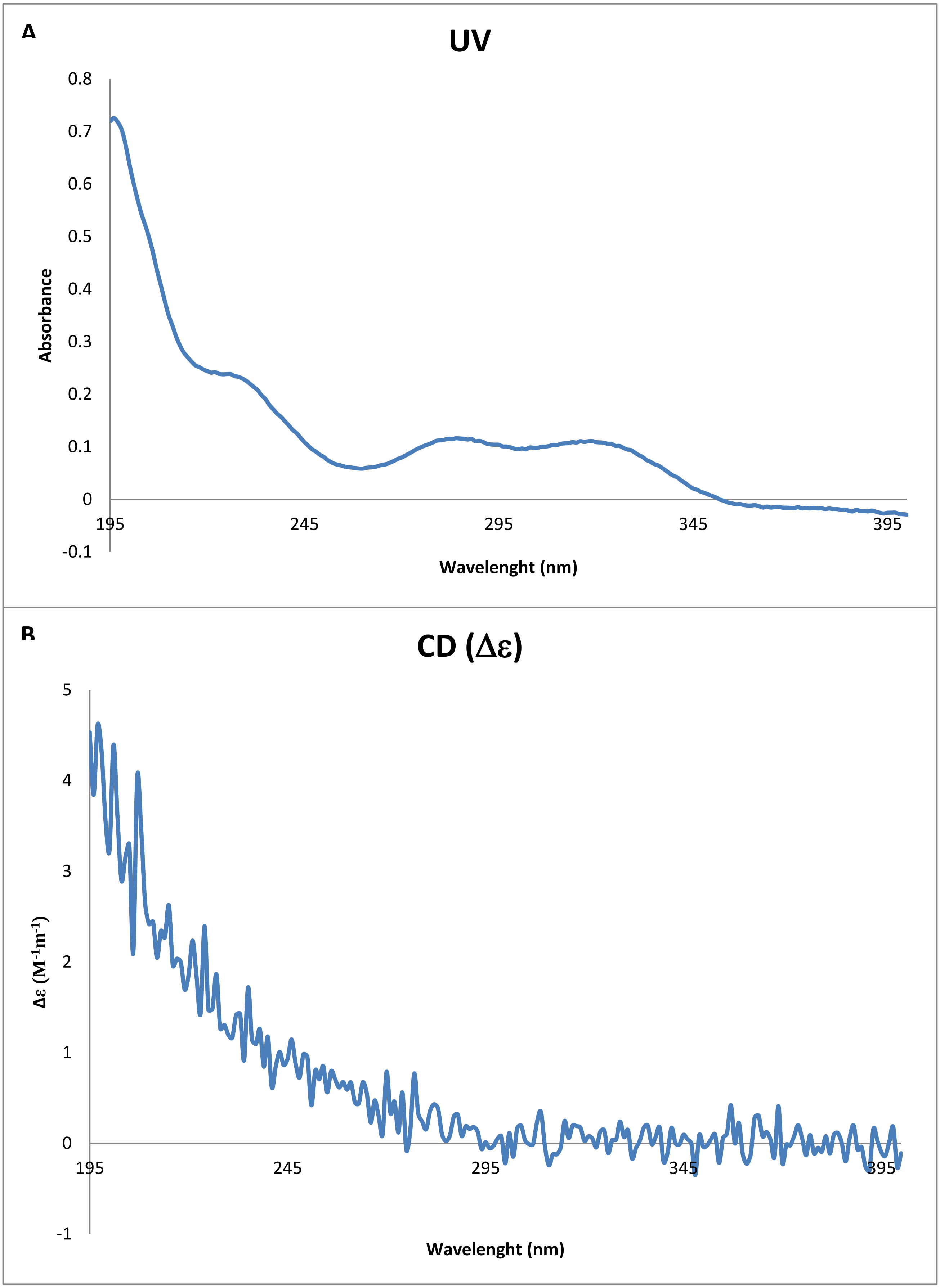


**Figure S2.** UV (**A**) and ECD (**B**) spectra for compound **9** in MeOH (0.07 mg/mL).

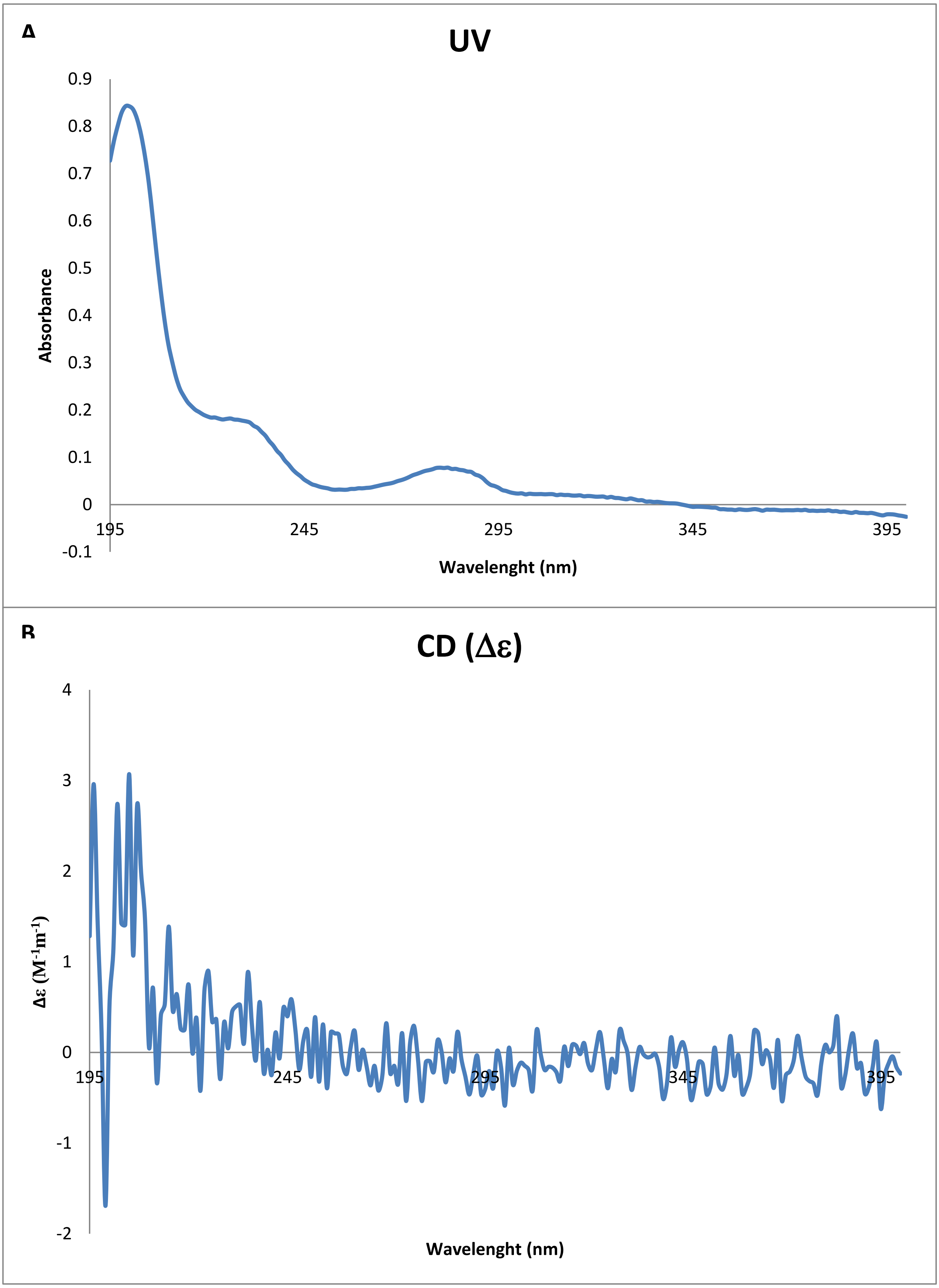


**Figure S2.** UV (**A**) and ECD (**B**) spectra for compound **10** in MeOH (0.05 mg/mL).

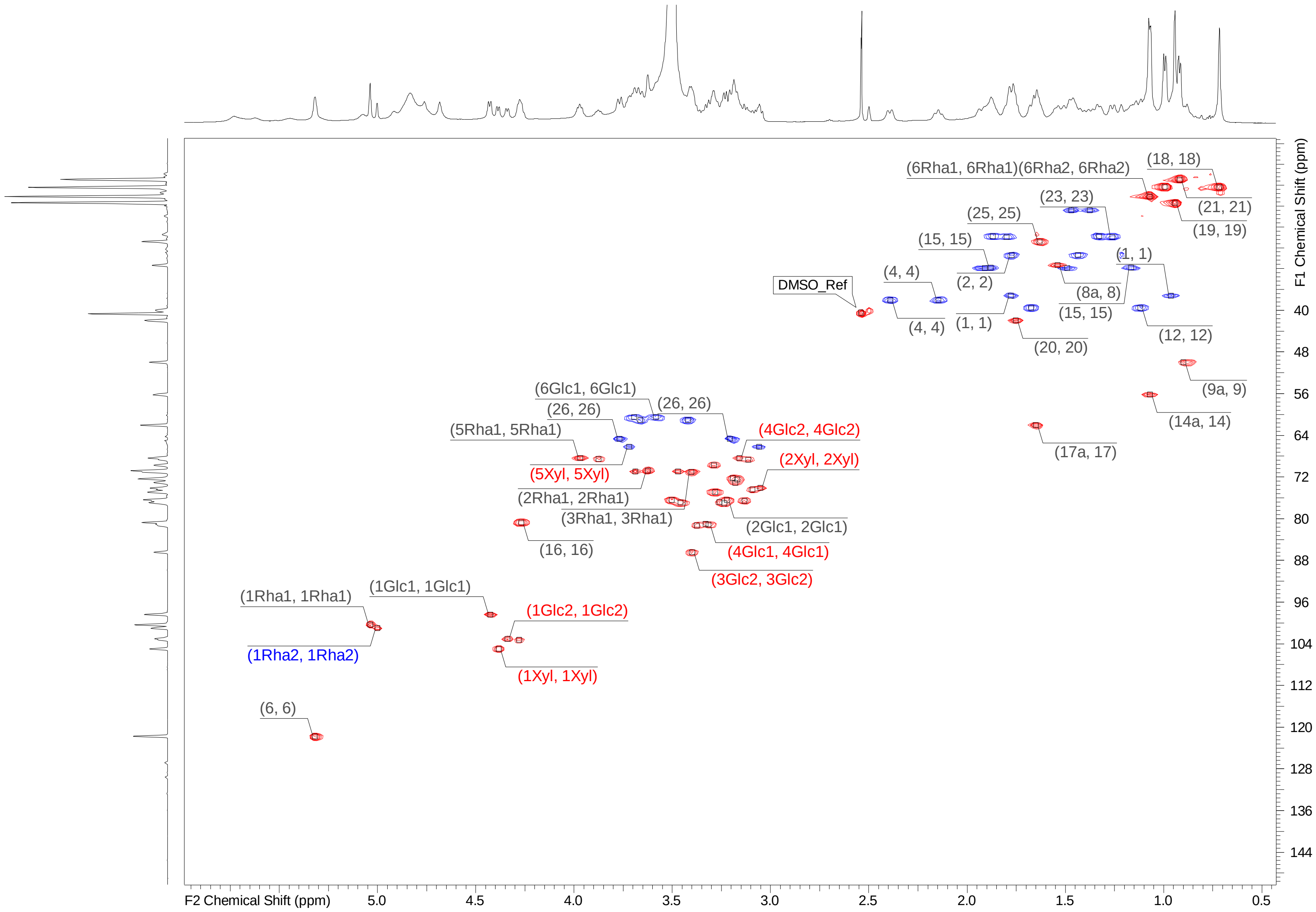


**Figure S3.** HSQC-DEPT spectrum of the mixture of compounds **11** (in red and black) and **12** (in blue and black) (500 MHz, DMSO)

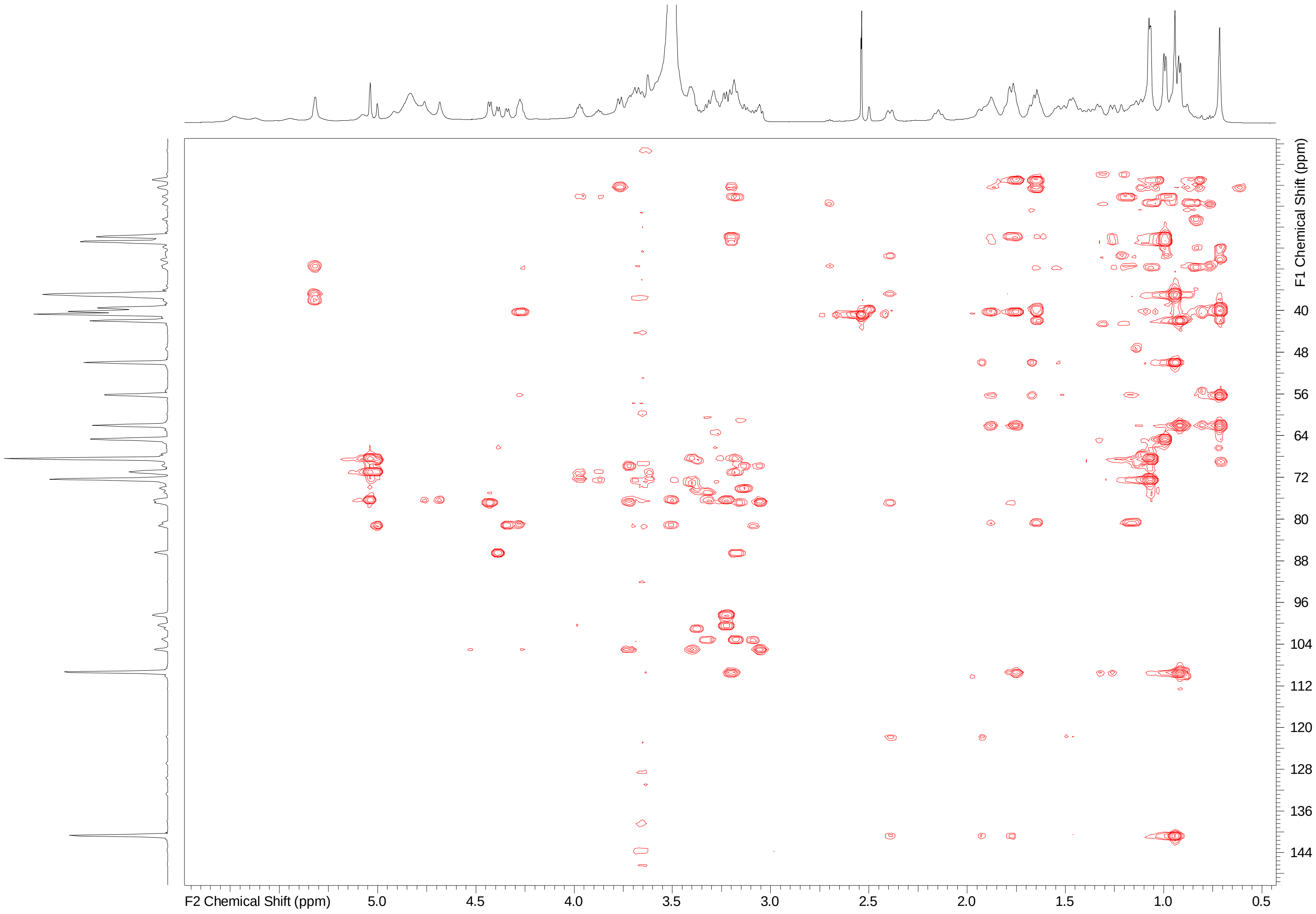


**Figure S4.** HMBC spectrum of the mixture of compounds **11** and **12** (500 MHz, DMSO)

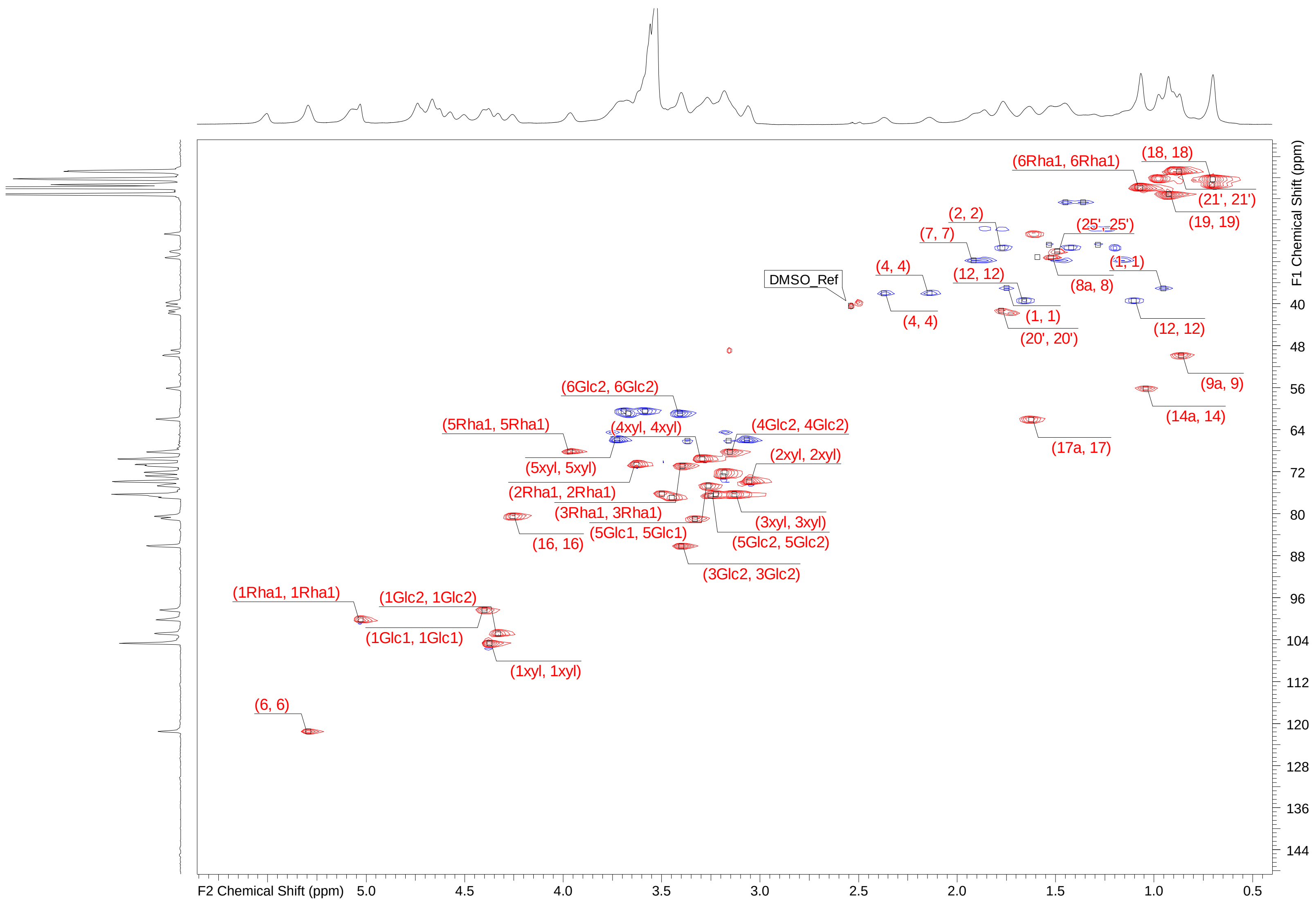


**Figure S5.** HSQC-DEPT spectrum of the mixture of compounds **11** and **13** (labelled in red, a prime was added for signals differing from **11**) (500 MHz, DMSO)

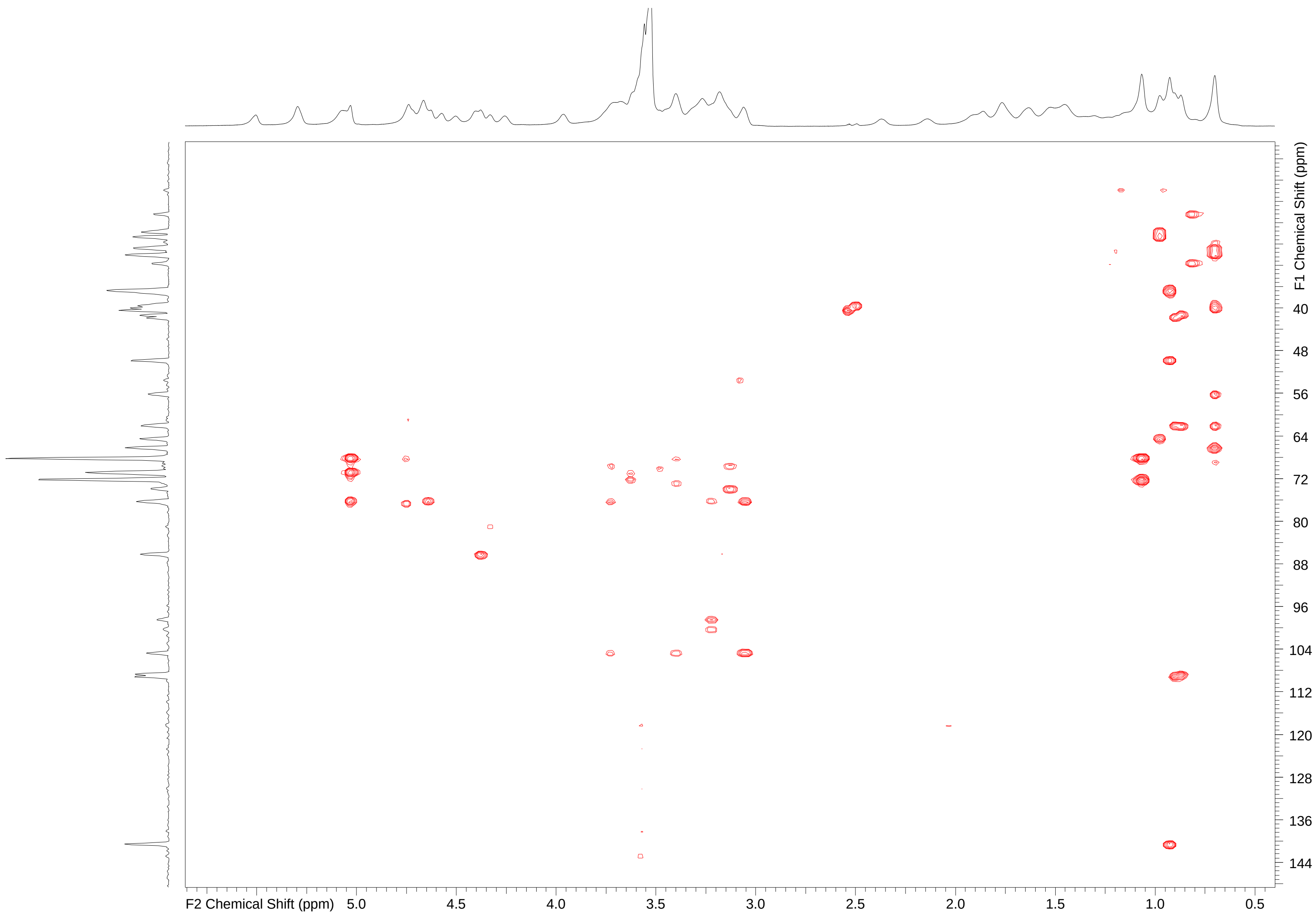


**Figure S6.** HMBC spectrum of the mixture of compounds **11** and **13** (500 MHz, DMSO)
